# Supplementary material for: Highly efficient CRISPR-mediated gene editing in a rotifer
Source: PLoS Biol. 2023 Jul 21;21(7):e3001888. doi: 10.1371/journal.pbio.3001888 (PMC10395877; doi:10.1371/journal.pbio.3001888)
Supplement: Attachment — Submitted filename: Response2Reviewers_20230501.docx [file pbio.3001888.s002.docx]

Reviewer #1:

Title: Development of Highly Efficient CRISPR-Mediated Gene Editing in the Rotifer Brachionus
manjavacas
Authors: Haiyang Feng et al.

Authors made CRISPR/Cas9-mediated knock out mutants with an application for knock-in technology by homology-directed repair using rotifers. Their success rate was 50-66% of the injected neonates survived to produce offspring. This is not bad if we consider rotifers are doing parthenogenesis.

However, the interesting researchers need to approach the genome information to determine off-target binding sites for Brachionus manjavacas. However, they are not published its genome into journal. This is a main flaw of this manuscript.

Response: We used the published genome GCA_018683815.1. We have moved the section describing this to be the second section of Materials and Methods so that it is more prominent.

The Discussion section is too short and they need to discuss more substantially to the better understanding for the readers.

Response: We have expanded the Discussion to put the results in context. However, as a methods paper we feel an extended Discussion section is not warranted.

Also in Fig. 2, the authors need to show HMA and % of mutant spectra of deleted mutants (should be reached to almost 100%) but they did not show it in the manuscript. 

Response: We do not believe that an analysis of the mutant spectra would be useful to the reader. The purpose of the manuscript is to describe a protocol that achieved CRISPR edited offspring with high efficiency. The specifics of the DNA repair mechanisms involved and the full repertoire of mutations that they could produce are outside the scope of this manuscript. As seen by comparing Fig2B and Fig2C the patterns of deletions are sequence-specific so an exhaustive analysis of these two genes is unlikely to be applicable to other targets.

Overall, I cannot support to publish this manuscript to PLoS Biology.

Response: We hope that the revisions to the manuscript address the reviewers concerns.


Reviewer #2:

The study be Feng et al., is a promising and exciting advance for CRISPR/Cas9 genome editing in the rotifer Brachionus manjavacas, an important animal system for examining molecular mechanisms of metazoan evolution. It will be of broad, significant interest to investigators using non-model system aquatic species for evolution and ecotoxicology, and more generally as another example of the universality of CRISPR/Cas9 genome editing across biological systems. The authors show robust targeted mutagenesis at two genes, vasa and mlh3, by injection of CRISPR/Cas9 RNPs in to the vitellaria of adult females. CRIPSR/Cas9 targeted knock-in of stop codon sequences from a single stranded oligonucleotide template was demonstrated at mlh3. The data indicates the edited alleles are transmitted through F1 animals to the F2 generation. Although CRISPR/Cas9 gene editing has previously been reported in somatic tissues of the rotifer Brachious koreanus using electroporation (Kim et al., 2019),
the current study by Feng et al., has very high novelty in demonstrating effective germline CRISPR/Cas9 gene editing and knock-in via injection into the vitellaria/oocytes of Brachionus manjavacas.

Overall the study was well organized but seems somewhat preliminary, and the presentation of how transmission of edited alleles is followed through the germline to the F1 and F2 generations could have been more clearly explained. **As presented the data does not demonstrate the establishment of stable, clonal mutant or precision knock-in lines, as claimed in the introduction**. In genetic model systems, a family of F3 animals is generated from a single F2 individual transmitting an edited allele, to establish a mutant line carrying the stable germline allele. **It was unclear if this is a feasible goal in this rotifer, since gene edited animals were inviable and infertile in the F1 and F2 generation**. T**he paper would be strengthened by establishing an F3 rotifer line that has a specific edit in the genome, and the line used for robust genetic analysis linking phenotype to genotype. If this is the longer term intent it could be better explained in the Discussion.**

Response: We apologize that our manuscript did not make it clear that we did establish several stable lineages of mlh knockouts and observed their phenotype over 20 generations. We have revised the section “mlh3 knockout phenotypes” to clarify this (vasa knockouts and many of the mosaic mlh knockouts did have reduced fertility).

Other issues:

1.      The very strong claims presented in the Discussion should be tempered to indicate further studies are needed to show indel and precision knock-in tagged alleles can be generated and used for mechanistic studies, and generation of reporter knock-ins is a possibility.

Response: We emphasize that we have demonstrated the generation of stable indel and knock-in alleles. We have qualified our conclusions concerning vasa and mlh3 function in the Discussion, as this was not the purpose of the manuscript.

2.      Other suggested improvements to strengthen the paper include optimizing targeted mutagenesis by titration of CRISPR/Cas9 reagents. Only one condition for CRISPR/Cas9 mutagenesis was tested, and only a percentage of the targeted animals survived.

Response: We agree that the protocol we present could be further optimized, and believe that it is most productive for this to be done by the community. Techniques may evolve to improve the 50% survival rate of injected mothers, though this will likely largely be a function of an individual’s experience with the technique. Given the very high frequency of edited offspring, and the fecundity of rotifers, even much lower survival rates would result in a researcher obtaining a mutation in a target.

There were resulting phenotypes including fertility and viability that were attributed to the targeted genes, but these phenotypes as indicated may be due to off targeting or gene knock-out by biallelic inactivation.

Response: As with any CRISPR experiment, off-target effects cannot be ruled out without additional experiments. The linking of phenotype to knockout was a secondary concern of the manuscript compared to demonstration that knock-out and -in editing can be obtained with high efficiency. However, we wish to note that in our limited examination of the phenotype of mlh3 knockouts, multiple independent knockout genotypes presented the same phenotype, suggesting knocking out mlh3 was the cause rather than multiple independent off-target effects. We agree that bi-allelic inactivation is responsible for the observed phenotype in some cases, which we describe as homozygous knockout.

Targeted knock-in of a 3Xstop cassette using an ssoligo template led to the identification of precise and imprecise alleles in F2 animals. Additional detail showing the lineage of alleles from each F1 would clarify the frequency of recovering precise integrations - not every F0 animal may transmit a precise knock-in allele. Having an idea of the number of F0 adults that need to be screened to recover a precise allele in the F1 to F2 generation is useful for replicating this approach.

Response: As described in the manuscript, half of injected mothers produced offspring with inserts, and from these about half of the F2 lineages had inserts, and about half of the inserts were precise. As noted in response to point 6 below, we have added the detail that all three of the tested mothers produced offspring with both precise and imprecise inserts. We have modified the Discussion to note that the exact frequencies for other inserts will vary depending on their sequence composition and length.

3.      Figure 1. The authors begin the results section with a clear description of the approach used for CRISPR/Cas9 gRNA RNP targeting of oocytes in the adult female vitellaria, and Figure 1 illustrates the approach. This is very much appreciated.

It would also be very helpful if there were a diagram of a flow chart of allele analysis through the F0 - F1 - F2 generations. A panel in Figure 1 showing life cycle or expected allele transmission (asexual or sexual) would be useful. A single F0 could transmit multiple alleles through the germline if the germline cells continue to be edited during early F0 development - this is established in other animal systems.

Response: We thank the reviewer for this suggestion and have added a diagram to Figure 1 with additional explanatory text in the figure legend.

Inclusion of the amount/molarity of reagents injected, either in the diagram, text, or figure legend would be helpful, particularly since this a novel approach for CRISPR/Cas9 targeting in rotifers. The methods state the concentration of reagents in the injection mix, but not the amount injected.

Response: We have added the volume and additional injection details to the Methods.

For future analyses, more consistent targeted mutagenesis may be achieved with synthetic guide RNAs from IDT or Synthego, which provide quality control, reducing possible toxicity due to the variable integrity of in-house synthesized and purified gRNAs.

Response: We thank the reviewer for this suggestion, which we have added to the manuscript.

4.      Figure 2 shows the results of indel targeting to vasa to generate lof alleles. In Figure 2B plot it was unclear what the y axis and x axis represent.  Is the x axis (1 - 11) the individual 11 mutant alleles shown in 2A? and the y axis is the number of times that allele appeared in individual F1s?

In Figure 2C, it seems there are 19 individual F1s represented on the x-axis. The y-axis is labeled mutagenesis ratio. The arrow pointing to Control - is this meant to be a value of zero? Many of the individual F1s noted on the x-axis show multiple alleles.

Response: We agree that these panels were not clear. We have revised both figures to emphasize the important findings: the relative proportion of individuals with each mutation listed in A, and for figure 3, the relative frequency of genotypes in each generation.

Pg. 7 lines 154-156: The text states "ongoing CRISPR activity carried from F0s to F1 oocytes or embryos" is responsible or F1s with 2+ alleles, and incorrectly cites references 24 (animal zygote targeting) and 25 (editing in arabidopsis). This is highly unlikely - CRISPR/Cas9 RNP particles would not be stable throughout the development of an F0 animal and segregated into its germline and F1 embryos. The citation # 24 by Mehravar et al., describes mosaicism in the F0 zygote targeted generation. Please remove this line and these references. F1 animals from a single F0 may inherit different germline alleles, but an individual F1 animal can only inherit, at most, two germline alleles.

Response: We have removed this sentence from the text. We agree that it is unclear how more than two mutant alleles arise in single individual F1s. This mosaicism could be caused by ongoing CRISPR activity carried from F0s to F1 oocytes or embryos given the small size and short generation time of rotifers. We now note in the Discussion that additional titration of injected reagents may reduce the frequency of mosaicism. We wish to emphasize that the goal of the manuscript is to provide a protocol for genome editing in Brachionus rather than to create specific mutant types. Given that mosaicism appears to be reduced with each successive generation, outcrossing of mutants is not needed in these asexually-reproducing rotifers, and phenotyping is easy and rapid, we believe the procedure presented here provides a significant advancement in the field.

The data indicated a stable germline vasa mutant couldn't be established since F1s died within 18 days. The text states this demonstrates a maternal effect, but to definitively show this, mothers that are homozygous for a mutant allele, generated by fertilization of haploid gametes carrying an established allele, is necessary.

Response: We agree that further genetic tests would be necessary to establish maternal effect, and have qualify our conclusion (“this suggests that vasa may be a maternal effect gene... However, additional focused studies will be necessary to support these preliminary observations.”

5.      Figure 3 shows the results of targeting the non-essential mismatch repair mutL Homolog mlh3. Panel 3D shows a mosaic F1 with overall reduced morphology and few oocytes, but the genotype of this individual isn't indicated. Again it isn't clear how an individual F1 could carry more than two alleles.

Response: The reviewer makes a valid point. Because this phenotype was not observed in any of the heterozygous double knockouts, homozygous knockouts, or the knockin mutants, we cannot make a conclusive link between a specific, mosaic, mutant genotype and the aberrant reproductive system phenotype. As the purpose of the manuscript is to demonstrate a protocol for CRISPR knockout and knockin gene editing rather than to explore this phenotype, we have removed this panel and any discussion of the implication of the phenotype, and simply report the observation in the results.

It could be helpful to target another gene not involved in DNA repair - disruption of mlh3 activity could have potential negative impact on genome replication during embryonic growth/cell divisions, leading to reduced viability and fertility. Targeting a developmental transcription factor involved in lineage specification, so that a stable line could be isolated, would be interesting.

Response: We observed no reduction in viability or fertility for the heterozygous double knockouts or the homozygous knockouts of mlh3 and have revised the manuscript to emphasize this. These knockouts produce the predicted phenotype. We agree that further pursuit of the role of mlh3 in rotifer reproduction would be interesting. The goal of the manuscript is to provide a protocol for genome editing in Brachionus rather than to investigate mlh3.

6.      Figure 4. F2 animals were recovered carrying precise and imprecise knock-in alleles of STOP codons, from an ss oligo template, at the mlh3 target site. From 6 injected mothers, 3 had F1 offspring transmitting, and F2 inheriting, knock-in alleles. A total of 18 F2s were genotyped - a record of which of the 3 mothers transmitted a precise allele to F1 and F2 would be useful to know. This would clarify how many F0 animals need to be raised to recover a precise knock-in allele transmitted to the next generation.

Response: We thank the reviewer for raising this important point. We have added to the manuscript that all three mothers that transmitted knock-in alleles produced F2 lineages with precise and with imprecise inserts. So while the exact frequencies may vary for other inserts depending on their sequence composition and length, it appears that about half the F0 offspring will carry a precise insert.

Reviewer #3:

This paper introduces an important new method that opens up functional genetics in the phylum Rotifera for the first time. Rotifers are used in lots of areas of research in part because they are small animals with fast generation times. I agree with the authors that current work is hampered by the lack of ways to alter genes to test their functional effects. Knowledge of the deep branching clade of animals that rotifers belong to is limited at present because of the lack of tractable genetic model systems. Two knockouts and one knock-in are presented.

One challenge due to the lack of previous tools is that good genetic/phenotypic markers (like eye colour in Drosophila) are not known already. Two genes with different presumed effects are chosen, and the primary method for gauging success is cloning and sequencing. It might have been interesting to see how expression level of vasa varies among the different mutant types to confirm that different amounts of the vasa gene are expressed depending on the pattern of mutation introduced. Some phenotypic observations are presented that fit with expectations, although both with a complication that wasn't predicted a priori (e.g. there is partial viability of vasa knockouts, irrespective of whether wt is present or not,; for mlh3 there is an apparent effect during amixis as well as during mixis). Is there any further evidence that can be used to confirm this is an unexpected effect of gene knockout as opposed to other unanticipated effects of the CRISPR manipulation (which might be continuing activity between generations)?

Response: As the reviewer notes, there are no defined phenotypes for gene mutations in rotifers. Definitely linking the more complex phenotypes discussed above to the knockouts of these two genes is beyond the scope of this manuscript, which seeks simply to establish an efficient protocol to generate knockout and knock in mutations.

In an ideal world, you might prefer clearer cut links from genotype to phenotype for the initial test of the method, but I think this encapsulates the severe challenges of the state of play in rotifer research. This manuscript paves the way for future studies that introduce gene reporters and systematically investigate other genetic pathways.

Response: We agree with this evaluation and hope that the method we present can pave the way for detailed functional studies of rotifer genetics, which are effectively impossible with some sort of transgenic technique.

Some more comments are listed below.

Line 75. It would be good to add half a sentence saying why affecting the germ line is key for the benefits outlined above.

Response: We thank the reviewer for the suggestion and have amended this sentence to “However, as with RNAi and CPP transfection, this method did not affect the germ line so did not result in a stable mutated lineage.”

Line 93. It might be clear to everyone, but worth highlighting these are all amictic still here?

Response: We definitely agree this would be worth highlighting and have added that each generation was produced asexually throughout the paragraph.

Fig 1C. I can't see much here except a bi-lobed red blob. Would it be permissible to highlight the outline of the animal to aid interpretation?

Response: We the thank the reviewer for the suggestion and have added the inferred position of the rotifer (which moved between the image in B and C so a direct overlay is not possible).

Line 112-115. I don't follow why it is useful to have a gene expressed in the vitellarium and oocytes, as your method is targeting DNA rather than RNA? Somehow it seemed to imply that it is good for the gene to be expressed in the place that you are injecting…? Also, you say it is expected to affect offspring viability - this seems an odd choice if you're wanting to detect transmission of the edit in the F1.

Response: In the complete absence of known phenotypes we chose vasa because the previous report of vasa mRNA localization allowed at least indirect inference of function. It actually has nothing to do with the location of the injection, and we apologize for introducing an obvious source of confusion. We have edited the paragraph to clarify this.

Line 159. If you think there was ongoing CRISPR activity, could it be that the decline was caused by other mutational events rather than haploidy of the vasa gene. For example, could your injected Cas9 recruit other RNA in the cells and cause different mutations?

Response: We cannot find evidence of CRISPR activity lasting as long as would be required for our hypothesis so have removed the preceding sentence. We doubt the injected Cas9 is recruiting other RNA given that we established stable lines of mlh3 mutants using the same protocol. (However we can’t rule out a sequence specific off-target effect from our vasa sgRNA so have also qualified conclusions about the effect of the vasa knockouts)

Line 190. Intriguing. Were any of these repeats close to your target region or are they far apart in the genome?

Response: Yes, very intriguing! None of the loci are particularly close to mlh3.

Line 235. Interesting additional effect of the manipulation. Is it 100% certain that 'amictic' egg production in Brachionus is really mitotic rather than automictic? (I imagine it is but mention just in case). Or again, could this be off-target residual effects of the Cas9?

Response: Asexual egg production in monogononts has always been interpreted as amictic, although a supplemental note in a recent paper about bdelloid reproduction challenges this traditional interpretation (Terwagne et al Sci Advances DOI:10.1126/sciadv.adc8829). Because we don’t have a good explanation for why this phenotype occurs in mosaics but not in stable double knockouts it may be some sort of off-target or residual effect, and we have removed any suppositions about revealing mlh3 function.

Line 261. Regarding the knock-ins, did you see any phenotypic effects of these?

Response: There was no reduction in viability or phenotype and males were not observed, but we did not maintain dense cultures over many generations to establish definitely that males were never produced.

Line 281 "Allows stable transgeneration lineages in about 3 weeks" - I thought they all became extinct after 18 days? Which cases survived for 3 weeks?

Response: We established clonal lineages of 6 double knockouts of mlh3 that we maintained for 10+ weeks (lines 216-220). We apologize that this was not clear and have revised this section.

A diagram of the design for each knockout and the knock in with number of individuals in F0 and F1 might be useful to have an overview of the design. I found it quite hard to keep track

Response: We thank the reviewer for this suggestion and have added a diagram to Figure 1 with additional explanatory text in the figure legend.
