# Supplementary material for: Highly efficient CRISPR-mediated gene editing in a rotifer
Source: PLoS Biol. 2023 Jul 21;21(7):e3001888. doi: 10.1371/journal.pbio.3001888 (PMC10395877; doi:10.1371/journal.pbio.3001888)
Supplement: Attachment — Submitted filename: Response to reviewers.docx [file pbio.3001888.s003.docx]

Response to reviewers:

Reviewer #2: The authors have addressed all issues.

This reviewer appreciates the author's explanation that an F0 could transmit more than two alleles to offspring. It makes sense, given the rapid development and short generation time of rotifers, and persistence of Cas9 RNP, Casa9 RNP could continue to mutagenize cells in the vitellaria and germline stem cells ovarium.

We thank the reviewer for reviewing the revised manuscript and we are glad that the revisios they suggested have made the manuscript more clear.
